# A Rank Model of Casting Non-Conformity Detection Methods in the Context of Industry 4.0

**DOI:** 10.3390/ma16020723

**Published:** 2023-01-11

**Authors:** Robert Ulewicz, Karolina Czerwińska, Andrzej Pacana

**Affiliations:** 1 Faculty of Management, Czestochowa University of Technology, Al. Armii Krajowej 19, 42-201 Częstochowa, Poland; 2 Faculty of Mechanical Engineering and Aeronautics, Rzeszow University of Technology, Al. Powstancow Warszawy 12, 35-959 Rzeszow, Poland

**Keywords:** mechanical engineering, quality management, non-destructive testing, checkpoint

## Abstract

In the face of ongoing market changes, multifaceted quality analyses contribute to ensuring production continuity, increasing the quality of the products offered and maintaining a stable market position. The aim of the research was to create a unified rank model for detection methods in the identification of aluminium casting non-conformities, in line with the paradigms of the fourth industrial revolution. The originality of the model enables the creation of a rank for the effectiveness of total inspection points allowing for the optimisation of detection methods. Verification of the model was carried out against the production process of aluminium casting. The model included the integration of non-destructive testing (NDT) methods and the analysis of critical product non-conformities, along with the determination of the level of effectiveness and efficiency of inspection points. The resulting ranking of detection methods indicated the NDT method as the most effective, which was influenced by the significant detection of critical non-conformities and the automation of the process. The study observed little difference in the visual inspection and measurement efficiency parameters, which was due to the identifiability of non-conformities with a lower degree of significance and the low level of inspection cost. Further research will look at the implications of the model in other production processes.

## 1. Introduction

Due to the turbulent environment of manufacturing companies and the ever-changing market, quality management (including quality control) has an important function in improving the products on offer and is thus one of the important factors in creating rules for manufacturing companies [1,2]. Quality control methods for aluminium castings include methods and diagnostic techniques from the non-destructive testing (NDT) group. These tests make it possible to recognise material inconsistencies, perform an assessment of material properties and measure the dimensions of objects, without generating changes in the functional properties of the diagnosed workpiece [3,4]. The implementation of this type of detection is necessary for the manufacturing industry to maintain an adequate level of quality and conformity of machinery, equipment and structures [5]. In particular, this is the case in aerospace [6,7], railway [8,9], oil and water [10], oil and gas [11], marine [12], nuclear [13], construction [14], automotive [15,16,17], as well as in the civil engineering [18,19] or timber industries [20]. This indicates that technological advances and user requirements have extended the application of NDT even to non-manufacturing-oriented industries, proving that these tests are not limited to anomaly detection only. This is one of the motives by which authors of scientific studies emphasise the importance of selecting an appropriate (in many respects) NDT method.

The eddy current method and the magnetic method are the most useful for the detection of planar non-conformities, mainly characterised by surface cracks, especially those oriented perpendicular to the direction of the magnetic field or the direction of eddy current flow [21,22]. The aforementioned methods facilitate the identification, but with a much lower degree of sensitivity, of spatial discrepancies (e.g., air bubbles) and only those located below the surface of the product [23].

The radiographic method shows considerable suitability for the detection of internal spatial discontinuities. Flat discontinuities can also be identified by this NDT method, however, only if they are located along the direction of radiation propagation [24,25]. Note that internal spatial discontinuities can be identified by the ultrasonic method [26].

The penetrant method [27] is also useful for detecting planar discontinuities of the nature of surface cracks. This method enables the detection of discrepancies arbitrarily located on the surface of products, also in the case of complex object shapes. The presented NDT method also allows the detection of certain types of spatial discontinuities (e.g., pores present on the surface of products) [28].

The penetrant method makes it possible to determine the severity and length of discontinuities [27,28], while the ultrasonic method determines the height, length and dimensions of discontinuities that are equivalent to certain reference discontinuities, as well as the severity of these discontinuities [4,5,12,26]. The radiographic method allows the determination of the length, height and severity of discontinuities [24,25], while the eddy current method allows the length, depth or height of the discontinuity and severity of the discontinuity [9,21,23], and the magnetic methods allow the characterisation of the discontinuity in terms of its length and severity [28].

Due to the issue of detection uncertainty that accompanies NDT methods and the specific technological capabilities of each method, literature studies propose the use of, for example, two consecutive methods [29]. However, the use of multiple NDT methods results in each successive NDT detection being an independent inspection point and a separate process. This situation results in a significant increase in inspection costs, increased inspection time and the involvement of additional staff to process the information obtained from the various inspection techniques. As a result, attempts are being made to combine methods at a single inspection point so that all key features extracted from the various NDT methods can be displayed on a single screen [30,31]. Work is also underway to develop new non-destructive inspection methods [32]. Research continues to be undertaken into reducing the preparation required, speeding up inspection and implementing inspection without interrupting work [33].

As companies move towards Industry 4.0, the demand for robots to perform non-destructive testing has increased. This trend stems from the global marketplace in which high-tech, robotics, artificial intelligence, unmanned automation, real-time digital imaging, 3D computed tomography and nanomicroscopy are taking shape [25,34]. Considerations for adapting NDT to automated realities have been made by many authors, who point out opportunities, barriers and opportunities for adaptation [35,36,37,38,39]. Fully exploiting and effectively integrating the potential of robotic systems is a significant challenge. It should be noted that parts of industrial robots or inspection tools are most often not dedicated to diagnostic and analytical systems [40]. Test automation is also considering the possibilities of applying IT principles to NDT in line with an industrial enterprise paradigm based on digitalisation, automation and robotisation [41].

Due to the multitude of possibilities for the realisation of quality control of aluminium castings using NDT methods, an important issue is the selection of effective methods in the context of the specifics of the product tested and the quality problems that accompany it [11]. The identification of suitable control methods usually involves the analysis of a considerable number of variables, in order to achieve the desired level of quality and organisational and financial benefits [25,29]. This issue highlights the importance of prioritisation and the relevance of using ranking techniques.

The proper definition of the ranks as well as the priorities of the differentiated situational components at the action preparation stage simplifies and unifies the decision-making process. These actions, simplify the consecutive activities and favour their economisation [42]. According to the literature, ranking organises and facilitates the resolution of issues that are related to the opinion of the actions carried out [43,44]. The particular importance of priorities and rankings is revealed when there are a large number of variable factors together with a large number of variable determinants [45]. Such situations are one of the characteristics of the functioning of modern manufacturing enterprises [46,47]. In issues characterised by complexity, ranking methods are useful.

One commonly used ranking method is that based on the 20–80 rule and the associated law of concentricity. This principle is clearly oriented towards selective thinking and acting by concentrating on the most important variables of the problem situation [47,48,49]. Based on an analysis of the literature, it can be seen that the 80/20 rule indicates that about 20% of the variables represent about 80% of the cumulative value of a phenomenon [50,51].

The authors [52,53] point out that the links between tasks, activities, processes or variables in general, as well as the type and strength of the relationship (and therefore the results of the analysis performed using a Pareto–Lorenz diagram) can be illustrated using a matrix diagram. This instrument is used to indicate the relationship that exists between two or more quantitative variables, without defining the direction of the possible relationship. The symbols inside the array indicate the strength of the relationship between the different elements [54,55]. This numerical technique is used in the field of organisation (e.g., training planning, marketing, sales) [53,55], data analysis (e.g., number of employees, number of orders per month) [52], and in the analysis of complex processes [54].

The analysis of the literature allows us to conclude that there is a research gap in ensuring the optimisation of inspection points by means of ranked quality control methods from the group of non-destructive methods within the entire production process of aluminium products.

The aim of the research was to create a unified model for the ranking of diagnostic methods and techniques, taking into account the assumed criteria, within the framework of the identification of non-conformities in aluminium castings, in accordance with the paradigms of the fourth industrial revolution. The method consists of integrating selected non-destructive testing and real-time analysis of the indications obtained by combining several interdisciplinary approaches into an integral methodological configuration that allows the level of effectiveness of individual inspection points to be determined and ranked.

The originality of the model lies in the fact that it not only supports the assurance of an adequate quality level of products but also allows the ranking of the importance of inspection points in the form of non-destructive testing, thus ensuring the efficiency of detection activities, the reduction of inspection time, the reduction of the need for human resources and the reduction of the total cost of inspection activities. A novelty is the sequential analysis referring not so much to the identified non-conformities in products, but to the selection of the checkpoint characterised by the greatest degree of efficiency in identifying critical defects in products, i.e., those which have the greatest impact on the loss of the desired quality level. The obtained efficiency ranking of control points allows the improvement of the production process and, at the same time, the improvement of any aluminium casting in accordance with technological possibilities and customer requirements.

## 2. Rank Model of Casting Non-Conformity Detection Methods

### 2.1. General Description of the Model

**Figure 1:**
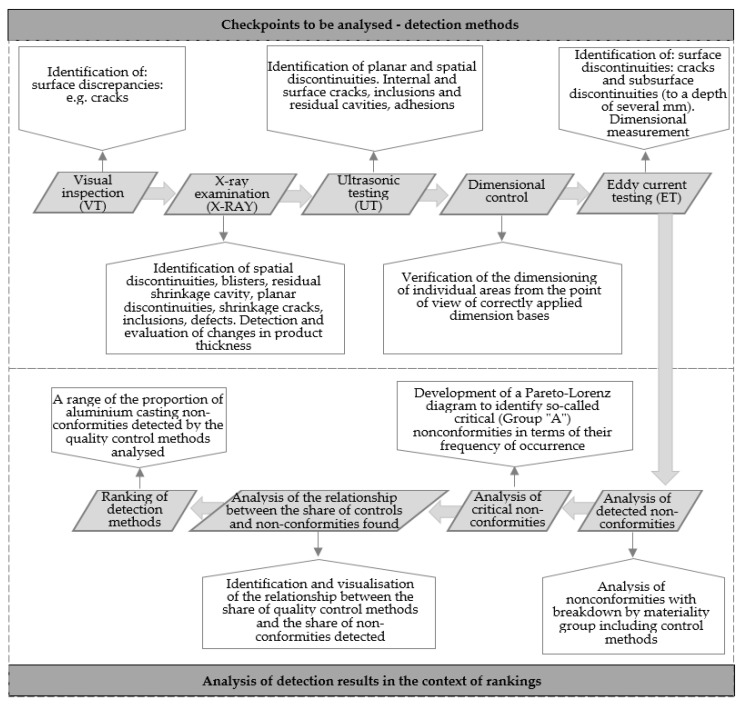
Overall model concept.

The model of a universal detection character through the identification of the most effective control points in the context of the assumed criteria (efficiency of the control point, cost and time of realisation of a unit detection) supports the quality management of the offered products in foundry companies. The developed model is based on a combination of non-destructive tests (visual, X-ray, ultrasonic, dimensional inspection, eddy current testing) located sequentially within the production process, analysis of the results from NDT tests and determination of the effectiveness of control points, determination of cost and time efficiency of detection realisation. This ultimately leads to an indication of the overall effectiveness parameter of the inspection points. Figure 1 shows a schematic concept of the model for casting non-conformity detection methods.

The model is divided into two areas: analysis of control points and analysis of detection results in the context of control point rankings. Thanks to the implementation of step control (diagnostic-analytical), the model allows going beyond passive control.

The rank model of non-conformity detection methods for aluminium castings is part of the trends of the fourth industrial revolution. Automated data analysis or matching of data from robotic detection with NDT enables the assessment of material properties and the provision of data for modelling and simulation of the behaviour of potential non-conformities [56,57]. The automation of detection testing significantly relieves the workload of employees, saving them time and energy (and thus costs). The use of mobile connectivity to transmit data from devices supports the digitisation process of the enterprise [40,58]. Within the framework of the proposed model, the possibility of automation refers in particular to the radiographic method, the eddy current method and the ultrasonic method.

The proposed model fits within the paradigms of the fourth industrial revolution. It can be seen reflected in the change in the human-machine relationship, i.e., the increase in collaboration between worker and robot, to which the development of intelligent interfaces contributes. Human labour will focus on managing the activities carried out by the sensing robot, which will lead to productivity gains. The paradigm shift from centralised to decentralised production also contributes to the development of NDT automation. This means the creation of intelligent process units and autonomous networks for information exchange and automatic configuration in order to achieve an optimal result (improvement of control points). The model developed is also in line with the paradigms present in the industry-market relationship (mass production, mass customisation and personalised production). Mass production, i.e., the manufacture of large quantities of identical products using specialised machinery, requires the use of fast and effective non-destructive quality control. The next paradigms—mass customisation, i.e., product variability and product personalisation—require a significant degree of flexibility in production and quality control, which can be provided by automated NDT.

### 2.2. Main Features of the Model

The guidelines for the usability of the model are mainly determined by the NDT methods used (their advantages, disadvantages and limitations in terms of implementation). The assumptions made were clarified after a literature review. In the implementation of the model, in the area concerning checkpoint analysis, the assumptions were:

- Availability of technological documentation,
- Availability of atlas of casting discrepancies,
- Availability of historical data,
- Availability of acceptance criteria,
- Possibility to use the model for products manufactured from electrically conductive materials,
- Possibility to use the model for products having an axisymmetric area in their geometry,
- The possibility to perform contact or non-contact eddy current defectoscopy,
- The possibility of automating radiographic, ultrasonic and eddy current defectoscopy,
- The assignment of a member of staff with the ability to interpret the test results,
- Provision of adequate lighting,
- Providing magnifying glasses and devices for remote observation,
- Providing a register of NDT test results.

Within the area concerning the analysis of detection results in the context of checkpoint ranking, it was assumed that:

- The analysis of the detected non-conformities takes into account:
  ○ The percentage of non-conformities, their classification (in terms of frequency of occurrence) into three groups: A—critical, B—significant, C—less significant, according to the principle of creating an ABC chart,
  ○ Assignment of the control method with the most frequent detection rate to each type of non-conformity,
- Analysis of critical non-conformities:
  ○ Graphical representation of groups of non-conformities takes into account a minimum of 10 defects,

No restrictions are indicated for the other stages of the model; their implementation is a consequence of the previous stages.

### 2.3. Detailed Description of the Model

**Figure 2:**
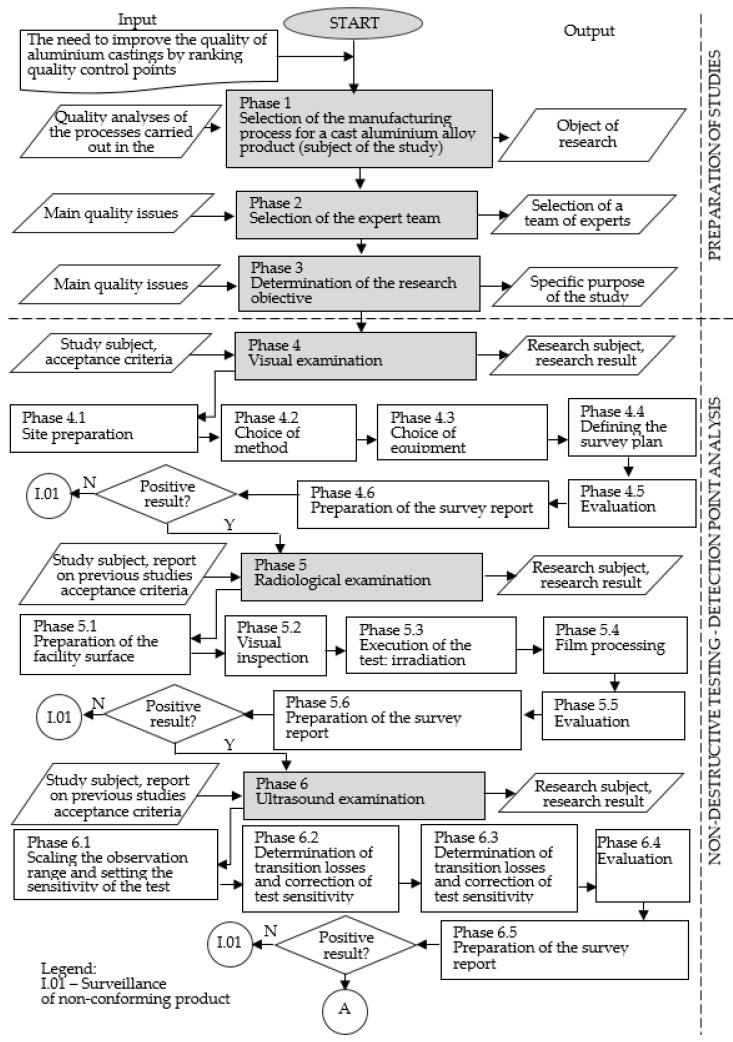
Rank model of casting non-conformity detection methods—part 1. Own development.

**Figure 3:**
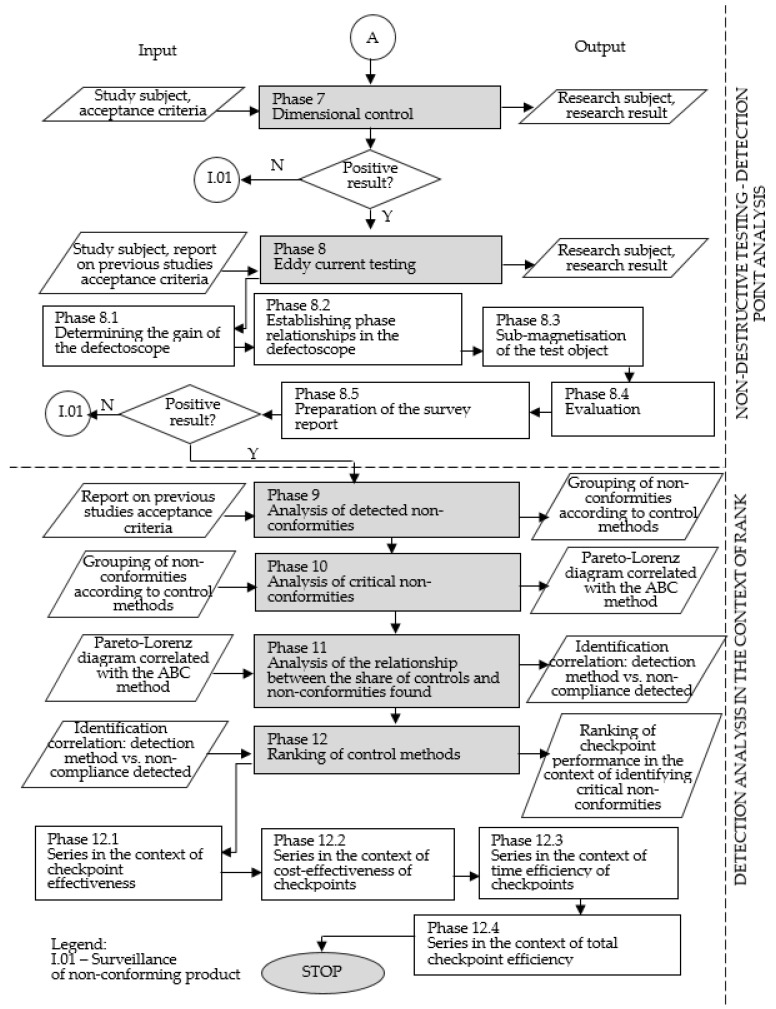
Rank model of casting non-conformity detection methods—part 2. Own development.

Once the assumptions of the model were taken into account, a detailed flow chart was developed. Figure 2 and Figure 3 show a description of the model including the input and output elements of the main phases of the model.

Phase 1: Selection of the manufacturing process for an aluminium alloy casting (test object)

The NDT tests used in the model condition the choice of test object (production process) resulting in an axially symmetrical aluminium alloy casting, which should be diagnosed in terms of control point rank.

Phase 2: Selection of the expert team

The selection of an appropriate team of experts is essential for the successful implementation of the developed model. The team of experts should have knowledge of the subject under study and experience in the use of NDT methods applied in the model, teamwork skills and competence to achieve the goal of the model implementation. The selection of the expert team members should be done according to the methods presented in the studies, e.g., [48].

Phase 3: Defining the research objective

The objective of applying the model should be to improve the process in terms of the quality of the manufactured products and process improvement, according to the Kaizen concept. In addition, the objective should refer to the elements that characterise the object of study.

Phase 4: Visual inspection

Visual inspection in the model is carried out as a preliminary visual inspection. The choice of the visual testing method (direct, indirect) is determined by the availability of the surface for visual inspection. Direct examination allows detection to be carried out with the unaided eye or using magnifying glasses and microscopes. Indirect examination is realised using endoscopes, videoscopes, periscopes and mirror sets. The choice of equipment mainly concerns the choice of illuminator and lighting conditions. The development of the examination plan concerns the determination of the course. Detected material discontinuities are classified on the basis of variables such as the number of occurrences, the type, size and severity of discontinuities. The detected irregularities are subject to marking. During the visual inspection, documentation must be drawn up describing the individual test results [59].

Phase 5: Radiographic examination

The radiographic method is used for testing materials: in corrosion detection, in object defectoscopy, in thickness measurements (including coating thickness), in micro defectoscopy of materials, and in microscopy, to determine the composition of materials. With this method, it is possible to detect spatial discontinuities, blisters and shrinkage cracks. It is also possible to ocean changes in object thickness and coating thickness [31].

Preparing the surface of the casting for examination involves removing the remnants of the moulding compound, reinforcement, cores and gating system. For this test, a sending transducer is placed on one side, which generates X-rays. The products being inspected absorb the radiation (gamma and X-rays) delivered from an external source. A detector (e.g., silver film, digital transducer, luminophore imaging plate) is placed on the other side of the device. The homogeneous radiation beam passing through the device is partially absorbed, which depends on the variation of the internal structure and is noticeable in the final image [31].

Radiographs show two-dimensional shadow images of three-dimensional discontinuities. The object discontinuities are in the form of darker areas, usually irregularly shaped located on a lighter object background. These images indicate the shape of the discontinuity and its dimensions (width and length) in a plane that is perpendicular to the direction of radiation propagation [33]. The dimensions (length and width) of the discontinuities are determined by direct measurement on the radiographs. Based on the length measurement of the discontinuity, the severity of the discontinuity is determined over a specific length section. It should be noted that the images of discontinuities seen on the radiographs are magnified in relation to the actual dimensions (geometric magnification phenomenon). The accuracy of the indication of the length and width of discontinuities is influenced by geometric relationships: the focal distance and the distance of the discontinuity from the film [34].

The test report should include: a description of the purpose of the inspection, a description of the product, the subject of the test, acceptance requirements, a description of the test method and technique, a description of the apparatus and the apparatus setting, an evaluation of the test results, the dimensions of the discontinuity and possibly the characteristics of the discontinuity, and an evaluation of the quality of the object in accordance with the requirements [31].

Phase 6: Ultrasonic testing

Ultrasonic testing has been used to identify internal, surface and subsurface discontinuities. The range of observation should be set so that the maximum path covering the entire area to be inspected is a maximum of 80% of the observation range. In this study, fundus echoes should be visible on the screen. Reference samples or standards are used to specify the sensitivity level of the detection. The limits of non-compliance registration should be adopted criteria in accordance with standards, technical conditions or other binding requirements [5,12]. At this stage, attention should be paid to the following parameters: wavelength, speed of propagation of the wave in the casting under test, speed of propagation of transverse and surface waves and transition losses.

Sensitivity correction is implemented due to transition losses. Accurate alignment of the camera and transducer is important, as it affects the correct imaging of the ultrasonic waveform in the test object and allows the depth of the discontinuity to be accurately determined [27].

As part of the evaluation and execution of the test, the displacement areas should be determined each time prior to detection, taking into account the test techniques, the beam insertion angle and the base material (minimum width 10 mm). During the detection, the test probe should be moved in such a way as to ensure the effectiveness of the detection of discontinuities and to obtain maximum information to determine their type and size. During the visual inspection, documentation indicating the individual test results should be prepared [20].

Phase 7: Dimensional inspection

Dimensional inspection involves inspection of the construction documentation, which involves checking the workpiece for dimensioning of key areas of the product.

Phase 8: Eddy current testing

Eddy current testing is used to inspect castings for discrepancies such as surface discontinuities, narrow-slit discontinuities and relatively large near-surface surface discontinuities [11]. High-frequency transducers are used to test the product’s surface layers. When investigating structural changes and discontinuities in the material located in a relatively shallow depth, low-frequency transducers should be used [21].

Sub-magnetisation of objects is realised in the case of automated testing of rods and tubes, manufactured from ferritic steels, using through-hole transducers [9].

Defectoscopy uses the phenomenon of eddy current relationships. The magnetic flux Φ induces an alternating electric field in the inspected product, causing eddy currents whose spread is disturbed by discontinuities. The eddy currents induce a magnetic field superimposed on the magnetic field induced by the coil. The inhomogeneities present in the material under test induce an uneven eddy current distribution and these induce an uneven resultant magnetic field [21,23].

According to recommendations from the literature on the subject and industrial practice, the eddy current test report should include the following information: description of the test subject, description of the test method and technique, acceptance requirements, description of the test equipment settings, evaluation and interpretation of the test result, characteristics of discontinuities, evaluation of the quality of the tested product. [23].

Phase 9: Detected discrepancy analysis

In order to carry out the analysis of the detected non-conformities in the applied control points, the following variables should be taken into account in tabular form: type of identified non-conformities, their percentage, cumulative value and their classification with allocation to groups in terms of frequency of occurrence (group A: critical non-conformities, B: significant non-conformities, C: less significant non-conformities) and in terms of the quality control methods within which they are identified.

Phase 10: Analysis of critical non-conformities

This phase is performed in order to visualise and analyse in detail the 10 most serious non-conformities on a Pareto–Lorenz diagram correlated with the ABC method, which allows prioritising the non-conformities influencing a significant decrease in quality of the manufactured batch. The graphical representation shows the relative and absolute distribution of the types of non-conformities, presenting the data in a column chart with the elements giving the greatest contribution to the problem highlighted.

Phase 11: Analysis of the relationship between share of control and detected non-conformities

The analysis of the relationship between the share of control and the detected product non-conformities should be presented using a matrix diagram. The diagram allows one to diagnose whether the analysed elements are related to each other and to determine the strength and type of relationship. The matrix diagram is divided into four areas, the points placed within them reflect the relationship that occurs between a specific detection method and the number of identified product defects detected by this method. The relationship can be labelled, e.g., strong, medium and weak. The correlation between non-conformities and the individual inspection methods in the study is examined in terms of effectiveness, testing time and testing cost.

On the basis of the identified correlation in the context of the detection performance of the control methods, the validity of the control points was determined, allowing a matrix diagram to be drawn up indicating the relationship between the effectiveness of a control point and the cost of implementing a unit test and the duration of the test.

Phase 12: Ranking of control methods

In this phase, the type of correlation is identified, which is based on ranks. Within the framework of the control method ranking model, the relationship between the frequency of non-compliance and the frequency of control methods is analysed in the first step. This relationship is understood as the effectiveness of the checkpoint expressed in percentage terms. This value is calculated from Formula (17).

(1) $$S=\mathrm{CN}\;\cdot \left(1-F\right)$$

where: S—checkpoint efficiency [%], CN—frequency of non-conformity detection [%], F—frequency of occurrence of the control method [%].

On the basis of the obtained values of the S parameter, an efficiency series is built (cost efficiency, time efficiency, total efficiency).

The next stage allows for the creation of a cost-effectiveness series. The cost-effectiveness values of individual control points are calculated from Formula (18).

(2) $$\mathrm{EK}=S\;\cdot \left(1-K\right)$$

where: EK—checkpoint cost efficiency [%], S—checkpoint effectiveness [%], K—unit detection cost [%].

Then, on the basis of Formula (19), the time efficiency is determined, which consists of checkpoint effectiveness and unit detection time. This parameter is calculated according to Formula (19).

(3) $$\mathrm{EC}=S\;\cdot \left(1-\mathrm{Cz}\right)$$

where: EC—checkpoint time efficiency [%], S—checkpoint effectiveness [%], Cz—unit detection time [%].

The last step is related to the calculation of the total efficiency of the checkpoints, which should be determined according to Formula (20).

(4) E=S·K·Cz

where: E—total efficiency [%], S—checkpoint efficiency [%], K—unit detection cost [%], Cz—unit survey execution time [%].

The obtained parameters of individual checkpoints (checkpoint effectiveness, cost-effectiveness, time effectiveness and total effectiveness) are plotted in the respective areas of the matrix diagrams. The diagram area distinguishes four fields specific to the variable under study. In each case, the most favourable area of the diagram is quadrant II—highlighted in grey.

The main idea of this phase is to rank the detection methods from the most effective, within the process, to the least effective, i.e., to rank the NDT methods.

The proposed model exploits the diversity and complementarity of different NDT methods and qualitative analyses to develop more effective synergistic systems. The model promotes a reduction in the level of diagnostic uncertainty through a stepwise approach to the analyses performed and an increase in the efficiency of quality control throughout the production process.

## 3. Model Verification and Results

The validation of the proposed model was carried out with reference to a selected production process in the framework in which quality stability was established. The process is realised in one of the foundry companies in the south-eastern part of Poland producing products for the energy, motorisation, medical and mechanical engineering industries.

Phase 1: Selection of the production process for an aluminium alloy casting product (subject of the study)

**Figure 4:**
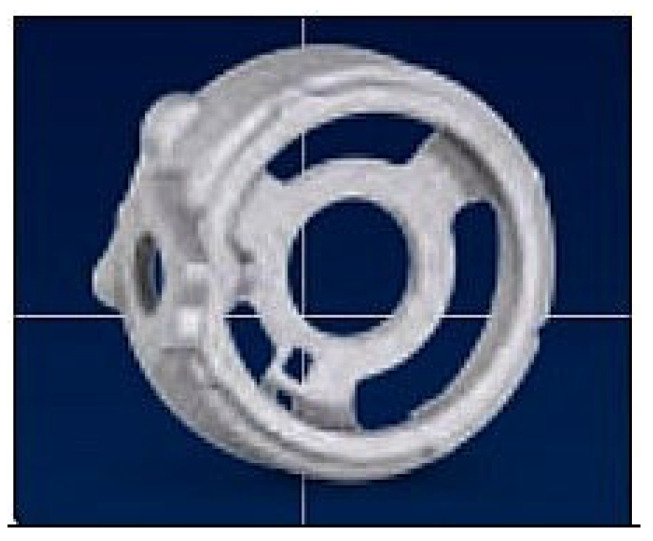
Adaptor flange model.

The test of the proposed model was realised for the production process of an adapter flange, which is used extensively in industrial automation. The product manufactured using the selected process was a critical point in terms of ensuring the desired level of quality of the company’s product range. The product was subject to design changes in accordance with the customer’s recommendations, with the consequence that the quality stability of the process was lost. A model of the adapter flange is shown in Figure 4.

The flange adapter, measuring 94 × Ø270 and weighing 2.6 kg, is cast by gravity from AlSi9Cu3 alloy.

Phase 2: Selection of the expert team

The second phase of the proposed method involved the selection of expert team members. The selection of the members of the expert team was made taking into account the specificity of the control points and the requirements related to knowledge and experience in the analysis of this type of quality problem. A team of experts was selected: a quality control manager, an NDT specialist, a quality control employee and product authors.

Phase 3: Defining the research objective

The purpose of using the model was to propose a way of analysing the effectiveness of the various checkpoints used in the quality control process of the adapter flange. By locating the checkpoint with the highest proportion of product non-conformity detection in the production process, it is possible to optimise the quality checkpoints, which will contribute to reducing the cost or time of production processes.

Phase 4: visual examination, Phase 5: radiographic examination, Phase 6: ultrasonic testing, Phase 7: dimensional inspection, Phase 8: eddy current testing

As part of the implementation of each stage of the production process, the product is subject to quality control. In particular, the verification concerns the technical parameters specified in the technological documentation of the product. Control of the adaptor flange production process is performed on the basis of the control plan. This plan specifies the types of control methods to be used, their distribution and frequency. Before carrying out the tests, it is necessary to determine basic information such as the scope of the inspection, the name of the product, the names of the measuring instruments, the method of measurement/verification, the expected values with parameter tolerances, the determination of the sample size, frequency, test stands, regulations and standards, as well as the skills and competence of the personnel carrying out the inspection. It is good to complete the control plan and procedure for dealing with non-conforming products.

The detection results from the individual checkpoints were the input for Phase 9.

Phase 9: Analysis of detected non-conformities

As part of the analysis of the control points and the correlation between the frequency of occurrence of each type of control during the detection of the adaptor flange casting and the proportion of non-conformities identified, a review of the detected non-conformities in the product produced by the investigated process over a period of 4 months was carried out. 269 adapter flange products were observed. A visualisation of the test results is shown in Table 1.

Table 1 denotes the quality control methods carried out at each inspection point in turn: X-ray—X-ray inspection, UT—ultrasound inspection, ET—eddy current inspection, VT—visual inspection and P—measurement (carried out as part of dimensional inspection). Table 2 also includes: KL—numerical inspection and KA—alternative inspection.

Phase 10: Analysis of critical non-conformities

**Figure 5:**
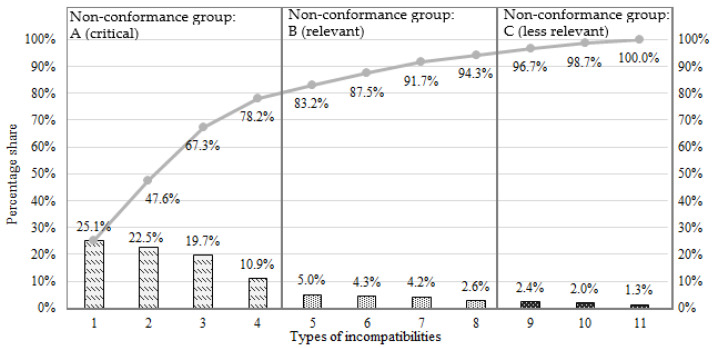
Pareto–Lorenz diagram of inconsistency.

As part of this step, a Pareto–Lorenz diagram was made, taking into account the ABC principle. The diagram is based on Table 1. The diagram made it possible to pick out critical non-conformities (in terms of frequency of occurrence). Figure 5 shows a Pareto–Lorenz diagram for the casting defects analysed.

The diagram adopts the same non-conformity designations as in Table 1. The critical non-conformities of the castings, from group “A”, are 4 non-conformities out of 11 listed, i.e., presence of shrinkage cavities, presence of oxides, presence of rows and internal cracks. These represent 78.2% of the quality problems identified. It is noted that defects classified as “critical” are identified using NDT—eddy current testing, ultrasound and X-ray testing.

Phase 11: Analysis of the relationship between the share of inspections and non-conformities detected

Table 2 refers to the next step of the analysis and contains data on the frequency and period of the checkpoint, as well as data indicating the determinations of the indicated defects by the methods analysed.

X-ray detection had the highest percentage of non-compliance detection among all methods analysed, at 44.8%. Visual inspection was the detection method that was frequently used (36. 4%).

Numerical inspection was involved in 18.2% of the non-conformity detection cases and detected 9.19% of all analysed non-conformities. Alternative inspection was used to detect 81.8% of non-conformities and detected 90.8% of non-conformities.

**Figure 6:**
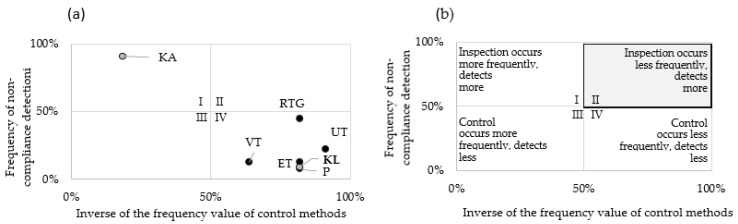
Matrix diagram showing (**a**) the relationship between the frequency of detection of non-compliance and the frequency of occurrence of a checkpoint in the context of its effectiveness (**b**) overview diagram.

The results of the relationship analysis (Table 2) were plotted on the corresponding boxes of the matrix diagram (Figure 6), produced in the next phase of the study.

Phase 12: Ranking of control methods

The area of the matrix diagrams used in this phase was divided into four sections. The grey colour indicates the quadrant in which the individual detection methods should ultimately be placed.

Figure 6a shows the relationship between the frequency of identification of a company’s key non-conformities and the frequency (incidence) of checkpoints. This relationship indicates the level of effectiveness of a given checkpoint. Figure 6b is an illustrative diagram to support the interpretation of the division of the matrix diagram areas. It is desirable to have a relationship with regard to control points: quality control occurs less frequently and detects more, as indicated by the second quadrant of the matrix diagram. In order to realise the objective function: the greater the product of the indicated values the better, the values of the function on the x-axis are expressed as (1-Frequency of control methods).

The control point analyses performed allowed the detection methods to be divided into the following groups (division according to the description of the quarters) in Figure 6b:

- Area I—quality control with a high percentage of detection of material discontinuities and relatively high importance of non-conformities: KA,
- Area II—quality control with a low percentage of detection of material discontinuities and relatively high importance of non-conformities: none
- Area III—quality control with a high percentage of detection of material discontinuities and relatively low importance of non-conformities: none,
- Area IV—quality control with a low rate of detection of material discontinuities and relatively low importance of non-conformities: KL, P, UT, ET, VT, X-ray.

Checkpoint performance parameters were developed using Formula (1).

(5) $$S_{P}=81.8\%\cdot 7.6\%=6.2\%$$

(6) $$S_{\mathrm{RTG}}=81.8\%\cdot 44.8\%=36.7\%\;$$

(7) $$S_{\mathrm{VT}}=63.6\%\cdot 12.9\%=8.2\%$$

(8) $$S_{\mathrm{UT}}=90.9\%\cdot 22.5\%=20.5\%$$

(9) $$S_{\mathrm{ET}}=81.2\%\cdot 12.2\%=10.0\%$$

Based on the effectiveness parameters, a range of control point effectiveness was drawn up according to the function the more the better.

RTG > UT > ET > VT > P(10)

According to series (10), the highest number of non-conformities (44.8%) are detected at the quality control point where the X-ray examination takes place. The high number of detected product non-conformities may be due to the low-quality level of the castings (quality control after the first production process of the product).

Based on the indications of the effectiveness of the individual checkpoints, it can be seen that the smallest difference between the effectiveness level of the checkpoints occurs between visual inspection and measurement—2%. A relatively small difference was also identified between the effectiveness of ultrasound and eddy current testing—10.5%.

**Figure 7:**
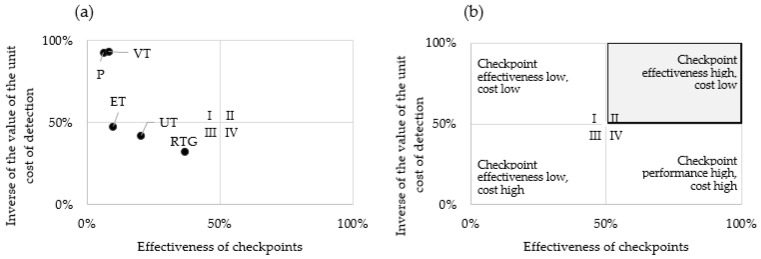
Matrix diagram showing (**a**) cost effectiveness of a given control point, (**b**) overview diagram.

**Figure 8:**
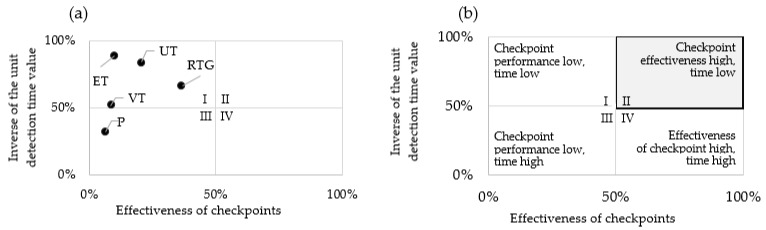
Matrix diagram showing (**a**) time efficiency of a given checkpoint, (**b**) overview diagram.

The series presented within Formula (10) is the input to the matrix diagrams for the analysis in the context of cost and testing time, as shown in Figure 7 and Figure 8, respectively. To realise the objective functions, the larger the product the better in terms of cost and detection time on the Y-axis, the parameters are expressed as (1)—analysed parameter, according to Formulas (2) and (3).

Figure 7 shows the cost-effectiveness of the checkpoint calculated according to Formula (2). Analogous to Figure 6a shows the result of the checkpoint analysis—the relationship between checkpoint effectiveness and unit cost of detection. Figure 7b indicates how to interpret the extracted quadrants of the diagram. The parameters for the effectiveness of the checkpoints were calculated as part of the earlier analysis step and the values for the unit detection costs were calculated on the basis of actual production data.

The checkpoint cost-effectiveness parameters were developed using Formula (2).

(11) $${\mathrm{EK}}_{P}=6.2\%\cdot 93.0\%=5.8\%$$

(12) $${\mathrm{EK}}_{\mathrm{RTG}}=36.7\%\cdot 31.9\%=11.7\%\;$$

(13) $${\mathrm{EK}}_{\mathrm{VT}}=8.2\%\cdot 93.4\%=7.7\%$$

(14) $${\mathrm{EK}}_{\mathrm{UT}}=20.5\%\cdot 42.1\%=8.6\%$$

(15) $${\mathrm{EK}}_{\mathrm{ET}}=10.0\%\cdot 47.7\%=4.8\%$$

Based on the values of the cost-effectiveness parameters, a series was drawn up with a function of the more the better (16).

RTG > UT > VT > P > ET(16)

According to series (16), the highest level of cost-effectiveness was identified at the X-ray inspection point, while the lowest was indicated for eddy current testing.

Analysing the indications of the cost-effectiveness of individual checkpoints, it was noted that relatively small differences in the examined parameter were found between ultrasound and visual inspection—0.9 per cent, and between measured inspection and eddy current inspection—1.0 per cent.

Figure 8 indicates the time efficiency of the control points calculated according to Formula (3). Figure 8a shows the relationship between the effectiveness of the checkpoint and the value of the unit detection time—checkpoint time efficiency. Figure 8b indicates how to interpret the quadrants of the diagram in the context of the variables analysed.

The time efficiency parameters of the control points were developed using Formula (3).

(17) $${\mathrm{EK}}_{P}=6.2\%\cdot 31.9\%=2.0\%$$

(18) $${\mathrm{EK}}_{\mathrm{RTG}}=36.7\%\cdot 66.6\%=24.4\%\;$$

(19) $${\mathrm{EK}}_{\mathrm{VT}}=8.2\%\cdot 51.7\%=4.3\%$$

(20) $${\mathrm{EK}}_{\mathrm{UT}}=20.5\%\cdot 84.3\%=17.3\%$$

(21) $${\mathrm{EK}}_{\mathrm{ET}}=10.0\%\cdot 88.2\%=8.9\%$$

Based on the values of the time efficiency parameters, a series was drawn up with a function of the more the better (22).

RTG > UT > ET > VT > P (22)

The interpretation of the diagrams in Figure 8 and the individual time efficiency parameters is analogous to the interpretation of the results of Figure 6 and Figure 7.

Analysing the indications of the time efficiency of the control points, it was noted that, also in this case, the X-ray control stands out with the highest parameter value. Relatively small differences in the parameter are found between eddy current inspection, visual inspection and measurement—they are 4.6 per cent and 2.3 per cent, respectively.

The X-ray examinations within the analysed process are performed in an automated manner, which significantly influences the nature of the ranking. The developed ranks and thus the time efficiency could be changed by upgrading the machine park, which would significantly contribute to reducing the unit detection time. All data relating to the implementation of X-ray quality control, checkpoint performance or detection results are available in real time and are further supported by augmented reality and optimised in an integrated network. This enables the adaptation of products to dynamically changing needs and the efficient use of raw materials, which, according to the literature [55], fits into the paradigms of the automated industry—the fourth industrial revolution.

The last step of the checkpoint ranking was related to the determination of the total efficiency parameter. This parameter takes into account the efficiency of the method, the execution time per unit test and the cost of the test). The aim of determining this parameter and then ranking the control points in terms of total effectiveness was to identify the most effective control method and the method with a low degree of effectiveness. This will enable effective and mature quality control management within the process. This parameter was calculated using Formula (4):

(23) $$E_{P}=6.2\%\cdot 7.0\%\;\cdot 68.1\%=0.2967\%$$

(24) $$E_{\mathrm{RTG}}=36.7\%\cdot 68.1\cdot 33.4\%=8.3372\%\;$$

(25) $$E_{\mathrm{VT}}=8.2\%\cdot 6.6\%\cdot 48.3\%=0.2614\%$$

(26) $$E_{\mathrm{UT}}=20.5\%\cdot 57.9\%\cdot 15.7\%=1.8594\%$$

(27) $$E_{\mathrm{ET}}=10.0\%\cdot 52.3\cdot 88.2\%=0.5847\%$$

Based on the values of the total efficiency parameters, a series was developed with a function of the more the better (28).

RTG > UT > ET > P > VT(28)

The high total efficiency parameter of the X-ray examination underlines its importance in quality control in the context of critical non-conformity detection and process automation. The presence of such non-conformities in the adapter flange casting is a major contributor to the quality stability of the production process. Due to the automation of X-ray detection, the overall efficiency rate is significantly higher in relation to the parameter indications of non-automated methods. This underlines the benefits of automated quality control.

The study also observed small differences in the total efficiency parameters of visual inspection and measurement, which is due to the relatively low level of inspection cost, the identifiability of non-conformities with a lower degree of significance (classified into area ‘C’ according to the methodology of the Pareto diagram correlated with the ABC method).

The developed model does not only contribute to the identification of critical non-conformities. With its participation, it is possible to monitor the level of effectiveness of control points located within the production process. The model allows the determination of cost, price and total checkpoint efficiency. The implementation of performance analyses allows you to identify a checkpoint that allows you to identify critical non-conformities—the most serious in terms of impact. Detecting the method characterised by the highest level of non-conformity detection and determining its position in the ranking of detection costs and testing time allows optimisation of the number and placement of inspection points. On the other hand, checkpoints with low detection rates as part of optimisation efforts can be converted to sampling. This action will make it possible to reduce the time and cost of quality assurance while maintaining a comparable level of meeting customer requirements.

The proposed model, based on integrally configured methods and analyses, makes it possible to go beyond passive control to analyses leading to improvements based on increasing the effectiveness of quality control in the production process under study, reducing costs and decreasing the total quality control implementation time by optimising the control system.

## 4. Discussion

The products offered by manufacturing companies must be tailored to customer requirements, as quality is a key criterion for the success of companies [1]. The cited concept of quality management in the literature is captured as quality assurance, which explicitly emphasises mature management focusing on advanced quality planning [3], improvement of product design [58], processes [60] and services [61], improvement of control in relation to processes and products [62], as well as explicit involvement of management in motivating employees [63]. In the literature, there are studies of research work in the field of pairwise comparisons of some quality control methods [64,65,66], comparisons of traditional control methods with their automated versions [67,68] and comparisons of more quality control methods [69,70]. However, there is a lack of studies comparing control methods in terms of their effectiveness in order to develop their ranking. For this reason, the aim was to develop a universal model for ranking detection methods through consistent analyses of the results obtained from unitary quality checks.

The model integrates quality control studies from a group of NDT methods and complex performance analyses leading to the ranking of detection methods in terms of effectiveness, cost-effectiveness and detection time effectiveness. By recognising the range of validity of inspection methods, it is possible to optimise the number and location of inspection points and to determine their adequate frequency (100% random quality control).

The model can be integrated with any number and type of detection method. The model was verified for the identification and analysis of adaptor flange casting non-conformities. In the case studied, critical non-conformities, identified according to the Pareto diagram and the ABC method, were found to be the presence of shrinkage cavities, oxides, ripples and internal cracks. Non-conformities were found by NDT testing. The ranking of the effectiveness of detection methods (visual, radiographic, ultrasonic, dimensional inspection, eddy current testing) indicated the X-ray method as the most effective, taking into account the frequency of identified non-conformities and the frequency of detection. Dimensional inspection—measurement was the least effective in the case study. In order to rank the total effectiveness of detection methods, in addition to effectiveness, cost and time of unit inspection were also taken into account. The total efficiency ranking indicated the X-ray method as the key quality control within the overall process. This result was influenced by the identification of non-conformities classified as key and the automation of the inspection process. The inspection points with the lowest total efficiency index were visual inspection, influenced by the traceability of casting non-conformities identified as ‘less relevant’.

The availability of a ranking of the overall effectiveness of checkpoints and the values of this parameter obtained by individual detection methods allows optimisation of quality control throughout the production process with the aim of reducing costs and production times.

It was observed that the degree of traceability of key product non-conformities and checkpoint automation, along with other benefits of computerisation, had the greatest impact on the total effectiveness parameter (taking into account the set variables) and thus the ranking position.

The values of the checkpoint ranking model take into account the occurrence of three main ranges of merit: the identification of equipment non-conformities throughout the production process, the level of content of the checkpoints and the compilation of a set of checkpoints in terms of their composition. The level of relevance was, for example, included: determining the level of diagnostic accuracy through the use of complementary NDT methods.

The limitations of the model are mainly determined by the NDT tests used in the model. The main limitation is the choice of product to be cast in aluminium alloys and the knowledge and experience of the expert team.

Future research directions will be concerned with the implications of the presented detection method ranking model for other manufacturing processes with low relatively low quality levels. In addition, it is planned to verify the model in other foundry companies applying NDT inspection to aluminium castings.

## 5. Conclusions

The model presented in the study for the ranking of non-conformity detection methods for aluminium castings enables the optimisation of control points within the entire production process. Quality management through the improvement of control points by means of automation, relocation and the use of selective control contributes to maintaining the stability of the production process.

The research that was carried out was aimed at developing a model to build the effectiveness of checkpoints in the context of their efficiency, the cost of survey implementation and survey time. The model developed consisted of three parts: test preparation, implementation of detection tests (NDT) and detection analysis in the context of rankings. The correlation occurring between the efficiency of the checkpoints and their cost and detection time was considered. This step allowed the total efficiency of the checkpoints to be determined and ranked.

The proposed model, based on integrally configured methods and checkpoint analyses, addresses not so much the identification of the critical causes of quality deterioration per se, but the issue of supervising the level of overall detection point effectiveness. This approach makes it possible to identify control methods that detect non-conformities of relatively high importance taking into account the cost and time of detection. This makes it possible to optimise them in line with the company’s preferences and capabilities. The calculation of the parameters indicated in the study facilitates quality management in terms of increased efficiency and flexibility of checkpoints. In addition, verification of the model indicated that rationally conducted quality control contributes significantly to ensuring production continuity. The obtained rankings of cost, time and total efficiency confirmed the positive significance of checkpoint automation. This conclusion fits in with the paradigms of the fourth industrial revolution.

Further research directions will be concerned with the implications of the proposed model for the pro-quality analysis of other production processes carried out in a foundry company, with the aim of ensuring an appropriate level of product quality and making cost optimisations and process time reductions. The proposed sequence of activities can be components of methods supporting quality management processes in foundry enterprises.

## Figures and Tables

**Table 1:** Non-conformities identified during the period under review.

Number	Type of Non-Compliance	Percentage Share	Cumulative Value	Quality Control Method with the Highest Detection Rate	Specification of the Type of Control
1	Presence of shrinkage cavities	25.1%	25.1%	RTG	KA
2	Presence of oxides	22.5%	47.6%	UT	KA
3	Presence of fissures	19.7%	67.3%	RTG	KA
4	Internal cracks	10.9%	78.2%	ET	KA
5	Dimensional discrepancies	5.0%	83.2%	P	KL
6	Edge splitting	4.3%	87.5%	VT	KA
7	Fogging	4.2%	91.7%	VT	KA
8	Misalignment	2.6%	94.3%	P	KL
9	Underfill	2.4%	96.7%	VT	KA
10	Scratches	2.0%	98.7%	VT	KA
11	Cracks	1.3%	100.0%	ET	KA

**Table 2:** Types and number of non-conformities of the adaptor flange casting during the period studied.

Detection Method	Frequency of Control Methods	Frequency of Non-Compliance Detection by Inspection Method
Dimensional inspection (P)	18.2%	7.6%
X-ray examination (XG)	18.2%	44.8%
Visual inspection (VT)	36.4%	12.9%
Ultrasonic testing (UT)	9.1%	22.5%
Eddy current testing (ET)	18.2%	12.2%
∑	100%	100%
Numerical control (KL)	18.2%	9.2%
Alternative control (KA)	81.8%	90.8%
∑	100%	100%

## Data Availability

Not applicable.
